# ‘Crystal Genes’ in Metallic Liquids and Glasses

**DOI:** 10.1038/srep23734

**Published:** 2016-03-31

**Authors:** Yang Sun, Feng Zhang, Zhuo Ye, Yue Zhang, Xiaowei Fang, Zejun Ding, Cai-Zhuang Wang, Mikhail I. Mendelev, Ryan T. Ott, Matthew J. Kramer, Kai-Ming Ho

**Affiliations:** 1 Hefei National Laboratory for Physical Sciences at the Microscale and Department of Physics, University of Science and Technology of China, Hefei, Anhui 230026, China; 2 Ames Laboratory, US Department of Energy, Ames, Iowa 50011, USA; 3 Department of Physics, Iowa State University, Ames, Iowa 50011, USA; 4 International Center for Quantum Design of Functional Materials (ICQD), and Synergetic Innovation Center of Quantum Information and Quantum Physics, University of Science and Technology of China, Hefei, Anhui 230026, China

## Abstract

We analyze the underlying structural order that transcends liquid, glass and crystalline states in metallic systems. A genetic algorithm is applied to search for the most common energetically favorable packing motifs in crystalline structures. These motifs are in turn compared to the observed packing motifs in the actual liquid or glass structures using a cluster-alignment method. Using this method, we have revealed the nature of the short-range order in Cu_64_Zr_36_ glasses. More importantly, we identified a novel structural order in the Al_90_Sm_10_ system. In addition, our approach brings new insight into understanding the origin of vitrification and describing mesoscopic order-disorder transitions in condensed matter systems.

Rapid solidification has been a promising materials processing technique to drive metallic systems out of equilibrium for the formation of amorphous glasses or composite materials with ultra-fine nanocrystalline meta-stable phases^1^. As-formed materials often display superior chemical or mechanical properties compared with their equilibrium counterparts^2^. While apparently lacking long-range translational symmetry, the undercooled metallic liquids and glasses have clear elements of short- and medium-range order^3^,^4^,^5^,^6^,^7^,^8^,^9^,^10^. A satisfactory knowledge of such structural order is essential not only to understand the glass formation, but to control the microstructures of the nanocomposite materials formed during rapid solidification or devitrification of as-quenched glasses^11^.

Various methods have been proposed to identify the short-range order (SRO) in non-crystalline metallic systems, ranging from pure geometric consideration^12^ to more sophisticated Voronoi tessellation^13^ and Honeycutt-Anderson (HA) common neighbor analysis^14^. So far, these methods have only recorded limited success: the former is based on the idea of dense random packing of hard spheres and often oversimplifies the chemical bonding in these systems^15^; while the latter is vulnerable to large deformations from an ideal motif due to the intrinsic amorphicity^5^,^16^,^17^.

Interestingly, none of the structural motifs uncovered in liquids or glasses are exclusive to amorphous structures; that is, they *can* pack into crystals. This is true even for the most commonly seen icosahedral order^18^,^19^,^20^,^21^,^22^,^23^, which contains non-crystallographic five-fold symmetry: numerous crystals contain nearly ideal local icosahedral ordering, and some of them are commonly used to model icosahedral quasi-crystals^24^. It was also demonstrated in a Lennard-Jones system that the SRO of the undercooled liquid matches that found in the crystal of the same composition^25^. Local packing is a relatively fast process compared with global networking of local clusters^5^; thus, even under rapid quenching, the system has sufficient time to survey the potential energy surface for a stable local packing motif. Given the energetic stability of the selected packing motif, it is possible that the same motif also appears in crystalline structures of close compositions. Such crystals may not be observed under actual experimental conditions. However, they should be accessible with a theoretical method that can efficiently search the configurational space for low-energy crystalline structures.

In this paper, we demonstrate the clear connection between crystalline and non-crystalline structural order in two systems: Cu-Zr and Al-Sm, representing strong and marginal glass formers, respectively. We first establish the dominant packing motif in crystalline structures identified by a genetic algorithm (GA)^26^,^27^, which is a robust means to locate low-energy configurations in crystals^28^,^29^. Then, we use the cluster alignment method^30^, which is analogous to the structural alignment used to identify regions of similarity in biomolecules^31^ to check the popularity of the GA-identified motifs in real undercooled liquids achieved by *ab initio* molecular dynamics (AIMD) simulations.

## Results

### Cu_64_Zr_36_ system

**Figure 1:**
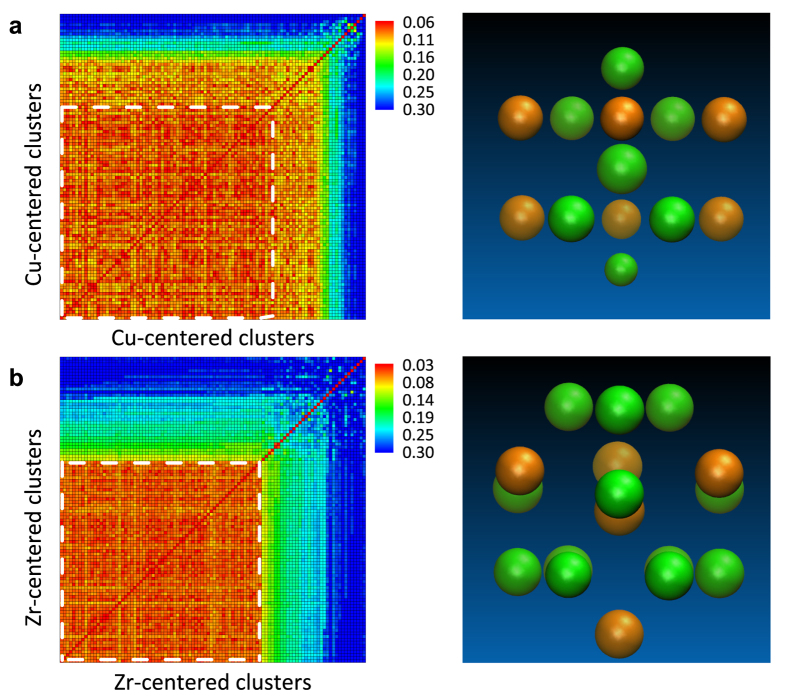
Pairwise cluster alignment for Cu-centered and Zr-centered clusters extracted from GA found Cu-Zr crystal structures. (**a**) The similarity matrix for the Cu-centered clusters. The matrix elements indicate the alignment score between two clusters. Smaller scores indicate higher similarity. The white dashed lines enclose a clique, that is, a submatrix whose matrix elements are all smaller than the cut-off value of 0.12. The right panel shows the icosahedral motif established by superposing the similar clusters, each pre-aligned against a fixed member of the same clique, and plotting an isosurface of high atomic density (see Methods) in the 3D space. Green and orange represent Cu and Zr atoms, respectively. (**b**) The similarity matrix for the Zr-centered clusters established in the same way as described in (**a**). The right panel is the superposed motif of similar clusters within the white dashed line, which gives a Frank-Kasper Z16 polyhedron.

We first revisit a well known glass-forming Cu-Zr system, focusing on one of the optimal glass-forming composition *x*_Cu_ = 0.64 32,33,34,35,36. The GA search was performed to collect low-energy crystal structures with unit cells containing up to 50 atoms and a Cu composition between 0.6 and 0.7. A semi-empirical potential in the Finnis-Sinclair form^37^ was used to expedite the GA search. The 100 lowest-energy structures found by GA were then collected for more accurate density functional theory (DFT) calculations as implemented in the VASP code^38^ (see methods). In order to identify the dominant packing motifs surrounding Cu and Zr atoms in these relaxed structures, we performed the cluster-alignment study on each pair of Cu-centered and Zr-centered clusters extracted from the relaxed structures. An alignment score was obtained to quantify the similarity between each pair of clusters (see methods). The lower the score is, the more similar the two clusters are. Figure 1a,b show the similarity matrix for the Cu-centered and Zr-centered clusters, respectively. The matrix element *M*_*ij*_ is the alignment score between the cluster *i* and cluster *j*. Similar clusters are organized into cliques. The lower left corners in Fig. 1a,b show the largest clique formed by Cu- and Zr-centered clusters, respectively. Here, a clique is a subset of clusters whose similarity matrix elements are all lower than a cutoff value of 0.12. Therefore, the clusters in each clique are expected to display significant similarity. The cliques in Fig. 1a,b contain 70% and 66% of Cu- and Zr-centered clusters, respectively, and thus should represent the dominant packing motifs. Indeed, once superposing these clusters, each pre-aligned against a fixed member of the same clique, one can see a distinct pattern in the atomic density of the superposed clusters, as shown in the right panels of the Fig. 1a,b for Cu- and Zr-centered clusters, respectively (see Methods for the calcultion of the atomic density of superposed clusters). The Cu-centered clusters give the icosahedral motif, consistent with a series of previous reports^16^,^39^,^40^,^41^,^42^. The Zr-centered clusters show the Frank-Kasper Z16 motif, which has also been reported before^16^, although in this study the dominance of the Z16 order can only be established through lengthy molecular dynamics simulations over hundreds of nanoseconds. These results clearly show that the two motifs, which are established in the GA-identified crystalline structures, are favored in both crystalline and amorphous structures of similar compositions.

Furthermore, other motifs such as distorted-icosahedral polyhedra (Voronoi indices <0,2,8,2>, <0,3,6,3> or <0,2,8,1>) and Frank-Kasper Z14 (Voronoi index <0,0,12,2>) and Z15 (Voronoi index <0,0,12,3>), which are found in CuZr liquid and glass state^43^,^44^,^45^,^46^, are also captured by GA-identified crystalline structures, as shown in the Supplementary Fig. 1. Although these motifs could also be a choice for local packing, the dominance of icosahedra and Frank-Kasper Z16 packing will eventually merge when given sufficient relaxation time^16^,^47^. Previous works^47^,^48^,^49^ also suggested interpenetrating icosahedral network as the origin of the medium-range order in this system. Such interpenetrating icosahedra are also observed in the GA-identified structures within relatively large unit cells. Please refer to the Supplemental Note 1 for more details.

### Al_90_Sm_10_ system

We have also applied this technique to reveal unknown structural order in undercooled Al_90_Sm_10_ liquids. As a member of the aluminum-rare-earth (Al-RE) series, this system can undergo deep undercooling and form amorphous solids or nanocrystalline composite materials with much improved mechanical properties than pure Al 2. Understanding the structure of undercooled liquids is critical for understanding and controlling phase selection in the design of a series of similar Al-rich materials.

Considering the large size disparity and affinity between Al and Sm atoms, we believe the Al_90_Sm_10_ system falls into the category in which the solute (Sm)-centered ordering plays the dominant role in defining the overall structural features^4^,^50^ (see Supplementary Note 2 for analysis of SRO around Al atoms). However, due to large coordination numbers (~16) of solute atoms and fcc-related packing in the system, even Voronoi tessellation analysis can hardly get a distinct polyhedron index for the system (see details in Supplementary Note 3). Thus, based on the concept of the crystalline gene in the liquid, we first identify extensive motifs in low-energy Al-Sm crystal structures with similar compositions, and then compare these motifs with the clusters in the liquid samples to establish the dominant SRO.

**Figure 2:**
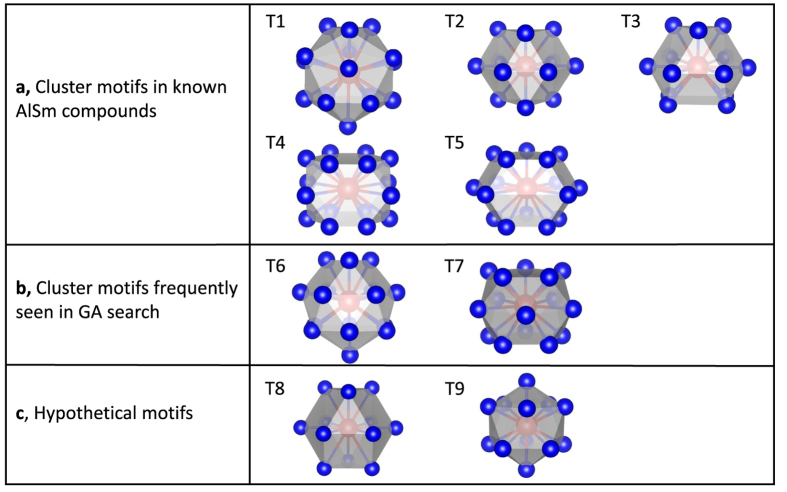
The Sm-centered template motifs containing the first atomic shell. (**a**) Template motifs extracted from known Al-Sm crystalline compounds^51^: T1, a typical C.N. 16 Frank-Kasper polyhedron, is extracted from the Al_2_Sm phase; T2 is extracted from Al_3_Sm; T3 from *γ*-Al_4_Sm; T4 from *β*-Al_4_Sm and *α*-Al_11_Sm_3_; T5 is another Sm-centered motif in *α*-Al_11_Sm_3_. (**b**) Two motifs frequently appearing in new crystalline structures found by GA search. (**c**) Two additional hypothetical motifs: T8 is the building block for fcc structures; T9 is an icosahedron. Red ball represents for center Sm atom and blue for neighbor atom.

As shown in Fig. 2a, the Al-Sm system has already shown a rich collection of Sm-centered ordering in known crystalline compounds, including Al_2_Sm, Al_3_Sm, Al_4_Sm and Al_11_Sm_3_, which have all been observed experimentally^51^,^52^. Since the composition of the target system (~10 at.% Sm) is different from any of the above compounds, we expect to see new structural motifs characterizing this composition range that are not covered in Fig. 2a.

**Figure 3:**
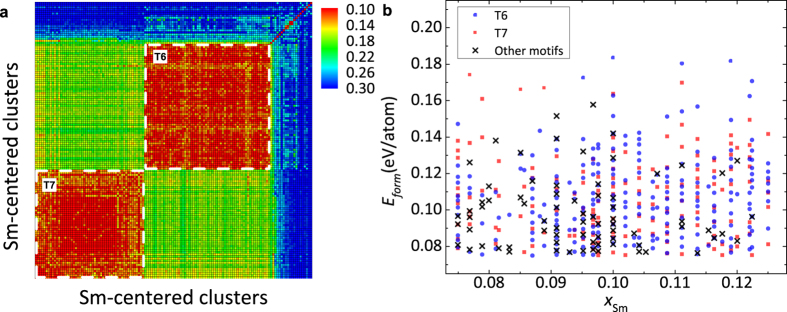
Similarity matrix and formation energy of low-energy Al-Sm structures found in GA search. (**a**) Pairwise cluster alignment for Sm-centered clusters in AlSm GA-searched crystal structures. (**b**) Formation energy as a function of the Sm composition, for a series of new phases found in the GA search. The structures containing T6 and T7 motifs are marked with circle and square, respectively. The crosses indicate the crystal structures without these two motifs.

To identify the missing motifs, we have performed GA search for low-energy crystal structures with unit cells containing up to 50 atoms over a narrow Sm composition range between 0.075 and 0.125. After performing pairwise alignment analysis similar to the CuZr system [see Fig. 3a], we found two new Sm-centered motifs, T6 and T7, as shown in Fig. 2b. The T6 motif consists of a top triangular Al layer followed by two hexagonal Al layers and a bottom Sm atom, whereas T7 contains three successive pentagonal Al layers. In Fig. 3b, we show the formation energy (*E*_form_), which is referenced to the stable Al_3_Sm and fcc Al phases, for ~500 structures with the lowest energies from our extensive GA search. These structures are all fully relaxed by DFT calculations. Again, the dominance of T6 and T7 motifs is clearly seen since structures containing these motifs cover the entire composition range, and comprise 50.3% and 33.5% of all the structures, respectively. The positive values of *E*_form_ show that these structures are unstable with respect to separation into Al and Al_3_Sm ground-state structures, consistent with the fact that the Al-Sm phase diagram shows no stable Al-rich compounds other than Al_3_Sm^52^. However, under fast quenching conditions, the pathway to phase separation into the equilibrium mixture of Al and Al_3_Sm can be kinetically by-passed, and local clusters of T6 or T7 can still be formed.

The preference of T6 and T7 will be checked against various other clusters in undercooled Al_90_Sm_10_ liquids. In addition to those existing in known crystalline compounds [T1–T5 in Fig. 2a], we introduce two more hypothetical competitors: T8 and T9 as shown in Fig. 2c. T8 is the building block of the fcc structure for pure Al, and T9 represents the icosahedral SRO commonly seen in amorphous structures.

**Figure 4:**
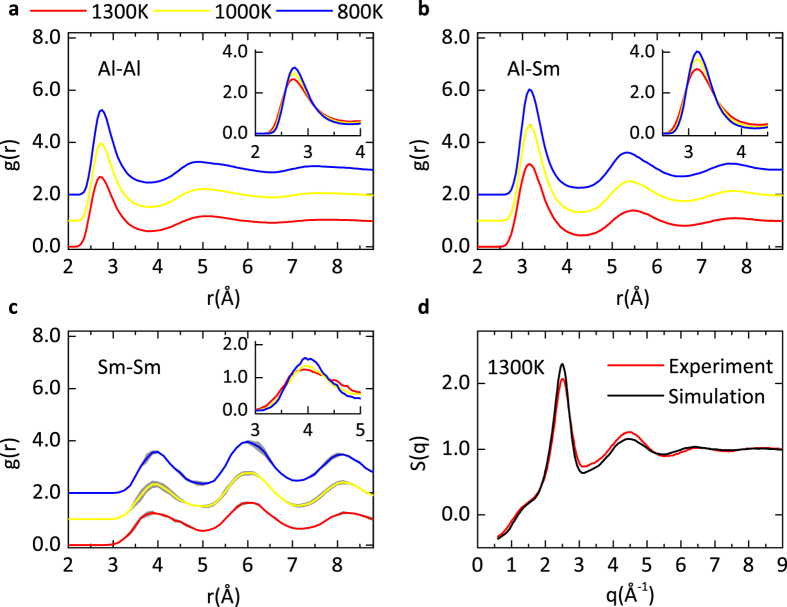
Validation of Al_450_Sm_50_ AIMD simulations. (**a**–**c**) The PPCF averaged over three AIMD Al_450_Sm_50_ samples for Al-Al, Al-Sm and Sm-Sm, respectively. The grey error band is generated by sweeping the error bar over all positions. The curves of different temperatures are shifted vertically for clarity. The inserts zoom in the first peak which is enhanced during the cooling process. (**d**) The structure factor obtained from AIMD simulations and experiments.

*Ab initio* molecular dynamics (AIMD) simulations (using VASP^38^) are performed to create three independent Al_90_Sm_10_ samples for a good statistical analysis (see methods). Each sample containing 500 atoms, which is large enough to fully capture the SRO, is annealed to equilibrate at 1300 K, 1000 K and 800 K. In Fig. 4a–c, the calculated partial pair correlation functions (PPCFs) of the samples are presented at various temperatures in the liquid or undercooled liquid regime. The PPCF is averaged over the three independently prepared samples. An “error band” is included by sweeping the error bar across all positions. The error bands for the Al-Al and Al-Sm PPCFs are vanishingly narrow. For the Sm-Sm PPCF, the error band is slightly broadened, since Sm is the sparse species in the system. Overall, Fig. 4a–c shows that the PPCFs for the three different samples are reasonably well converged, and thus the structural features extracted from these samples are statistically valid. Furthermore, we calculated the total structure factor *S*(*q*) of the simulated samples at 1300 K following the technique proposed in ref. 53. As shown in Fig. 4d, the calculated *S*(*q*) compares favorably with that measured in X-ray diffraction experiments^54^ (see details in Supplementary Note 4), confirming that our simulations reliably capture the structural properties of the Al_90_Sm_10_ system.

**Figure 5:**
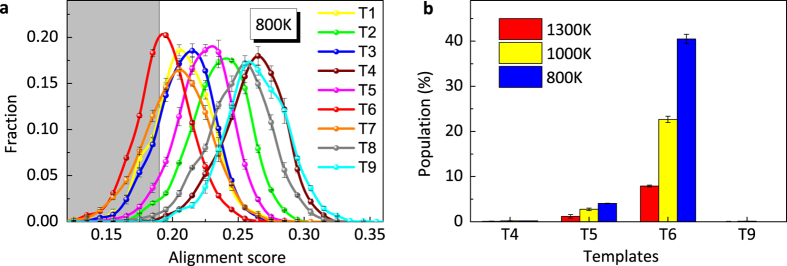
Short range order around Sm atoms by cluster alignment. (**a**) The score distributions for different template motifs aligned with as-extracted clusters from samples at T = 800 K. The gray region indicates the score is less than the cut-off value 0.19. T1–T9 indices correspond to the template motifs in Fig. 1. (**b**) Populations of four typical template motifs in AIMD samples obtained by the template-assisted cluster alignment

We first independently align the Sm-centered clusters extracted from the AIMD samples against each template motif shown in Fig. 2. Figure 5a shows the distribution of the alignment score for all template motifs aligned with the clusters at *T* = 800 K, where one can see vastly different peak positions for different templates. The alignment score reflects the similarity of the as-extracted clusters to the template motif; thus, the GA-identified motif T6 has the highest popularity since it shows the leftmost peak position as shown in Fig. 5a.

A closer inspection of the template motifs in Fig. 1 shows that T6 also shares similarity with a group of other motifs: T1, T2, T3, T7 and T8, while the remaining motifs T4, T5 and T9 are well separated from this group (see details in Supplementary Note 5). In Fig. 5b, we show the population of four topologically distinct motifs T4, T5, T6 and T9 in AIMD samples at several temperatures, using T6 to represent the group with similarities. Here, the motif with the lowest alignment score is used to characterize an as-extracted cluster in AIMD samples. However, if the lowest alignment score is larger than a cut-off value of 0.19, which is close to the peak position for T6 in Fig. 5a, the cluster remains uncharacterized. The temperature varies from above the melting point of 1253 K^52^ to deeply undercooled regime. The averaged population over three independent samples is shown along with the error bar. At *T* = 1300 K, less than 10% Sm-centered clusters from the liquid samples can be characterized with the four typical motifs, due to poorly developed local structural order at this temperature. Among them, the T6 motif already has a considerable population of 8%. With the temperature decreasing, the unknown population reduces, indicating the enhanced SRO. The same trend can be seen from the increment of the first peak height in the PPCFs during the cooling process, as shown in the inserts of Fig. 4a–c. At *T* = 800 K, the total population of identified clusters increase to 40%, most of which belongs to the T6 motif. This clearly shows that the T6 motifs, commonly seen in Al-Sm crystalline structures with *x*_Sm_ close to 0.1, are also characteristic of undercooled amorphous structures with similar compositions, while other three motifs are essentially nonexistent in the samples.

## Discussion

**Figure 6:**
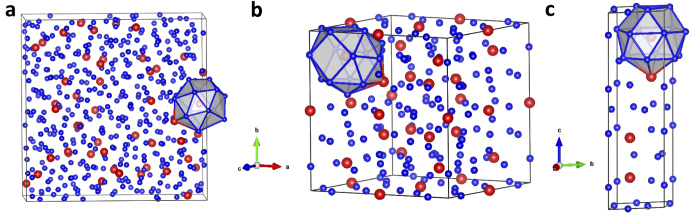
The commonly shared T6 motif in AlSm liquid and crystal structures. (**a**) The T6 packing motif around Sm atoms in simulated Al_450_Sm_50_ undercooled liquid sample at 800 K; (**b**) The T6 motif in Al_120_Sm_22_, the Al-Sm big cube phase, which is the first observed crystal structure from the devitrification process of Al_90_Sm_10_ samples prepared by melt spinning; (**c**) The T6 motif in Al_20_Sm_4_, which is one of the first devitrified phases from Al_90_Sm_10_ samples prepared by magnetron sputtering.

Experimentally, Al_90_Sm_10_ glass samples can be synthesized by different techniques, such as melt spinning and magnetron sputtering. When thermally annealed, the samples prepared by different methods (e.g., liquid processing vs. sputtering) can exhibit much different devitrification behaviors, which lead to different dominant crystalline phases in the first devitrification step^55^,^56^. Nevertheless, both crystals share the same T6 motif surrounding Sm atoms as the undercooled liquids in our AIMD simulations^56^,^57^, as shown in Fig. 6. The similarity of the SRO between the crystal structures and the amorphous parent structure can probably lower the transformation barriers between the glass phase and crystal phase, which may be the reason why these complex metastable phases appear first in the devitrification process. The different devitrification pathway for melt-spun or sputtered samples is likely attributed to differences beyond SRO in these samples, which is an intriguing subject for future studies.

In summary, we developed a systematical scheme integrating genetic algorithm, *ab initio* MD simulations and the cluster alignment method to reveal crystal genes, that is, the dominant SROs that transcend metallic crystals, glasses and liquids. With two glass forming systems, we show that the SROs characterizing low-energy crystals also have abundant population in deeply undercooled liquids or glasses with similar composition. By establishing the connection between crystalline and non-crystalline orders, our work provides a systematic approach to address a key question in determining the prevailing forms of non-crystalline order in liquids and glasses.

## Methods

### Adaptive Genetic algorithm

We use a genetic algorithm to search for low-energy crystal structures and obtain favored packing motifs for CuZr and AlSm systems. Based on the conventional GA scheme^26^, a classical potential in the Finnis-Sinclair^37^ form is employed in the adaptive GA to quickly calculate energy during the GA search. A portion of low-energy structures obtained in the GA search are then collected for accurate calculations using the density functional theory (DFT). The DFT results are used to adapt the parameters of the auxiliary classical potential. The above process is repeated until the structures collected in the DFT calculation pool are converged.

### Density functional theory (DFT)

All DFT calculations are performed using the Vienna *ab initio* simulation package (VASP)^38^. The projected augmented-wave (PAW) method is used to describe the electron-ion interaction, and the generalized gradient approximation (GGA) in the Predew-Burke-Ernzerhof (PBE) form is employed for the exchange-correlation energy functional.

### *Ab initio* molecular dynamics simulations

We simulated undercooled liquid samples of Al_90_Sm_10_ with *ab initio* molecular dynamics simulations. The constant number of atoms, volume and temperature (NVT) ensemble is applied with Nose-Hoover thermostats. The Verlet algorithm is used to integrate Newton’s equation of motion, using a time step of 3 *fs*. Three different samples, all with 450 Al atoms and 50 Sm atoms, are created independently for better statistical analysis. To construct these samples, randomly generated configurations with cubic supercell are equilibrated at 2100 K over 2000 time steps. Then each sample is cooled down to 800 K, well-below the melting temperature 1253 K with a cooling rate of 2.2 × 10^13 ^K/s. After that, the structures at 1300 K, 1000 K and 800 K are collected separately for further isothermal annealing for about 6,000 time steps. The first 3,000 time steps are not used in the analysis to ensure equilibrium has been reached.

### Cluster alignment methods

To evaluate the similarity between two independent cluster motifs, we employ a cluster alignment algorithm, following the “Individual cluster-template alignment” in ref. 17. We take one cluster as a fixed template, and align the other cluster to it. An alignment score, describing how the aligned cluster deviates from the template, is defined as


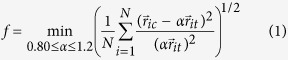


where *N* is the number of the neighbor atoms in the template; 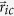 and 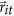 are the atom positions in the aligned cluster and template, respectively; and *α* is a coeffient to adapt the template’s bond length. The range of *α* is between 0.8 and 1.2, which allows a large enough “breathing” room for the bond length in templates, in order to achieve an optimal alignment. The smaller score indicates a higher similarity between the two clusters.

To reveal the common SRO in the clusters that belong to the same clique, we align all the clusters against a fixed member of the same clique, and superpose the aligned clusters by overlapping their centers. A continuous 3D atomic density of the superposed clusters is calculated according to


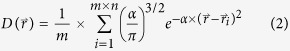


where 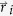 is the position of atom *i, m* is the number of superposed clusters, and *n* is the number of atoms per cluster. Here, the atomic density of a single atom is smeared by a Gaussian distribution. An isosurface of 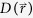 is plotted in the right panel of Fig. 1.

## Additional Information

**How to cite this article**: Sun, Y. *et al*. ‘Crystal Genes’ in Metallic Liquids and Glasses. *Sci. Rep.* **6**, 23734; doi: 10.1038/srep23734 (2016).

## Supplementary Material

Supplementary Information
