# Supplementary material for: ‘Crystal Genes’ in Metallic Liquids and Glasses
Source: Sci Rep. 2016 Mar 31;6:23734. doi: 10.1038/srep23734 (PMC4814814; doi:10.1038/srep23734)
Supplement: Supplementary Information [file srep23734-s1.pdf]

Supplementary Information for "'Crystal Genes' in Metallic Liquids and Glasses"

Yang Sun[1,2], Feng Zhang[2†], Zhuo Ye[2], Yue Zhang[2], Xiaowei Fang[1,2], Zejun Ding[1], Cai-Zhuang Wang[2,3], Mikhail I. Mendelev[2], Ryan T. Ott[2], Matthew J. Kramer[2], Kai-Ming Ho[1,2,3*]

[1]Hefei National Laboratory for Physical Sciences at the Microscale and Department of Physics, University of Science and Technology of China, Hefei, Anhui 230026, China

[2]Ames Laboratory, US Department of Energy, Ames, Iowa 50011, USA

[3]Department of Physics, Iowa State University, Ames, Iowa 50011, USA

To whom correspondence may be addressed: † fzhang@ameslab.gov or * kmh@ameslab.gov

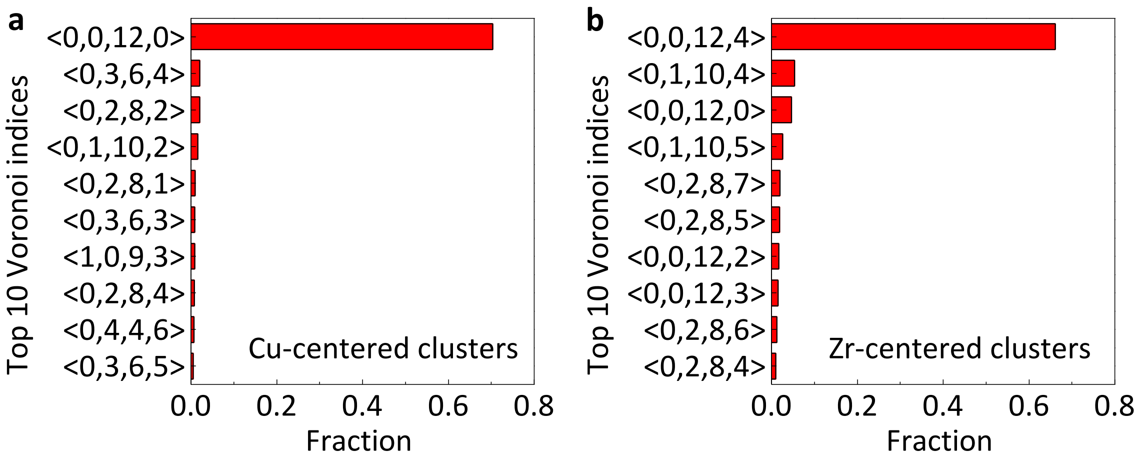

Supplementary Figure 1 | Voronoi indices of top ten polyhedra in GA-search Cu-Zr crystalline structures. (a) The top ten Voronoi polyhedra for Cu-centered clusters in GA-searched low-energy structures. (b) The top ten Voronoi polyhedra for Zr-centered clusters in GA-searched low-energy structures. The distorted icosahedral motifs (<0,2,8,2>, <0,3,6,3> and <0,2,8,1> polyhedra) for Cu-centered clusters, as well as Frank-Kasper Z14 (<0,0,12,2> index) and Z15 (<0,0,12,3> index) for Zr-centered clusters are all captured in the GA search with very small probability of occurrence in the crystal structure.

Supplementary Note 1: The interpenetration between icosahedral clusters characterized by GA-searched Cu-Zr crystal structures. Previous works suggested interpenetrating icosahedral network as the origin of the medium-range order in the Cu-Zr glass system [1, 2, 3]. Here, we examined the GA-identified structures with relatively large unit cell (containing 41-50 atoms/unit cell), and did see a strong interpenetrating icosahedral network. Supplementary Figure 2 shows the pair distribution function (PDF) of the *centers* of the icosahedral clusters identified in these structures. The PDF is averaged over all the structures considered. One can see a strong first peak around r = 2.5

Å. This peak is contributed by interpenetrating icosahedron pairs, in which the center of one icosahedron belongs to the first shell of the other. It shows a good agreement with the icosahedral PDF of $Cu_{64.5}Zr_{35.5}$ glass [3], indicating a common interpenetrating icosahedral network in both glass and GA-searched low-energy structures.

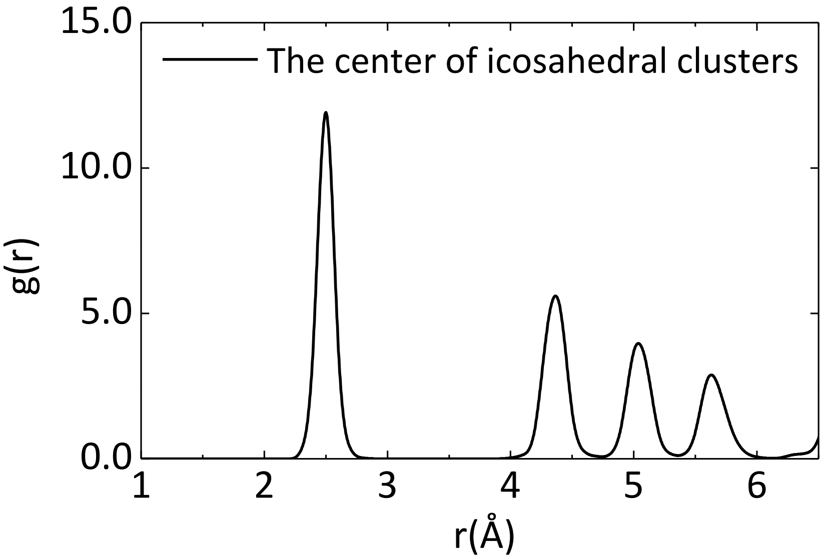

Supplementary Figure 2 | The pair distribution function of the centers of the icosahedral clusters identified in GA-searched low-energy structures.

Supplementary Note 2: Short range orders (SRO) surrounding Al atoms. We analyzed the clusters surrounding Al atoms in the crystalline structures obtained in the GA search. In structures with very low Sm composition, there are "pure Al" regions which displays f.c.c. ordering. An example is given in Supplementary Fig. 3. In Al-centered clusters that contain one or more Sm atoms, we failed to identify any dominating motif, which is in sharp contrast to the Sm case. Previous studies [4, 5] using hypothetic icosahedral and f.c.c. template motifs to investigate SRO around Al atoms in AlSm liquid samples only find small populations (~5%). On the other hand, the dominating T6 motif comprises 40% of solute Sm-centered clusters. The fact that the

local order surrounding Al atoms is much less pronounced than that surrounding the solute Sm atoms indicates that the $Al_{90}Sm_{10}$ system falls into the category in which the SRO of the system is characterized by solute-centered clusters [6, 7].

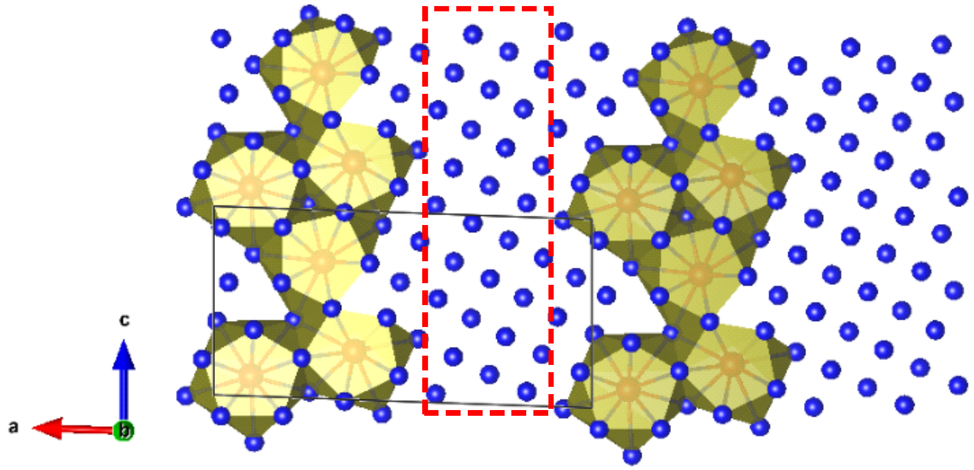

Supplementary Figure 3 | One example for pure Al region in the crystal structure searched by genetic algorithm. The chemical composition of the crystal structure is $Al_{37}Sm_3$. The black box indicates the unit cell. The red ball stands for Sm atoms, while blue for Al atoms. The Sm-centered clusters are shown with golden polyhedra. The dashed box shows the "pure Al" region that displays fcc ordering.

Supplementary Note 3: Voronoi tessellation for Sm-centered clusters in $Al_{90}Sm_{10}$ system. The characterization of structural ordering in the $Al_{90}Sm_{10}$ system with the help of GA-identified motifs cannot be achieved in the conventional Voronoi tessellation analysis. Supplementary Fig. 4a gives the 10 most frequent Voronoi polyhedra surrounding Sm atoms in the amorphous samples at T = 800 K. One can see a widespread distribution of these polyhedra. The most popular polyhedron with an index <0, 1, 10, 6, 0> only has a population of 8%. All these polyhedra have large coordination numbers of

16 or more. Consequently, small distortions can result in dramatic change of the Voronoi tessellation indices. Moreover, it has been demonstrated that Voronoi indexing on clusters that carry close-packing features such as fcc or hcp are particularly vulnerable to distortions [8]. For these reasons, the Voronoi index corresponding to the ideal T6 motif, <0, 3, 12, 1, 0>, only carries a vanishingly small population. In Supplementary Fig. 4b, we show the distribution of the 10 most frequent Voronoi polyhedra surrounding a sub-group of Sm atoms that have been characterized to be T6-type by the cluster alignment method described in the main text. Interestingly, this distribution highly resembles the one for the entire set of Sm atoms shown in Supplementary Fig. 4a. This demonstrates that in Voronoi tessellation, this sub-set of Sm atoms is treated as if they were randomly selected from the whole sample. In other words, the order characterization achieved by GA and template-assisted cluster alignment is not reflected in the Voronoi tessellation at all.

We also made a comparison of the top ten Voronoi indices between the 40% T6-type clusters and the clusters that failed to be characterized by any known motifs (~55%) in Supplementary Fig. 4b and 4c, respectively. The two distributions are both widespread, and share all but four types of Voronoi polyhedra (the indices for common Voronoi polyhedra are shown in red). It indicates the Voronoi index can totally confuse the structural topology. For instance, we find two topologically different polyhedra with same Voronoi index <0,1,10,5> in the GA-searched Al-Sm crystal structures named <0,1,10,5>-α and <0,1,10,5>-β as shown in Supplementary Fig. 4d and 4e, respectively. One can see that the <0,1,10,5>-α polyhedra is very close to T6 motif except one neighbor atom distorted. However, the packing of <0,1,10,5>-β polyhedra is very far from T6 motif. Thus, we can reach a similar conclusion that the Voronoi tessellation

cannot reflect the orders characterized by the GA and template-assisted cluster alignment.

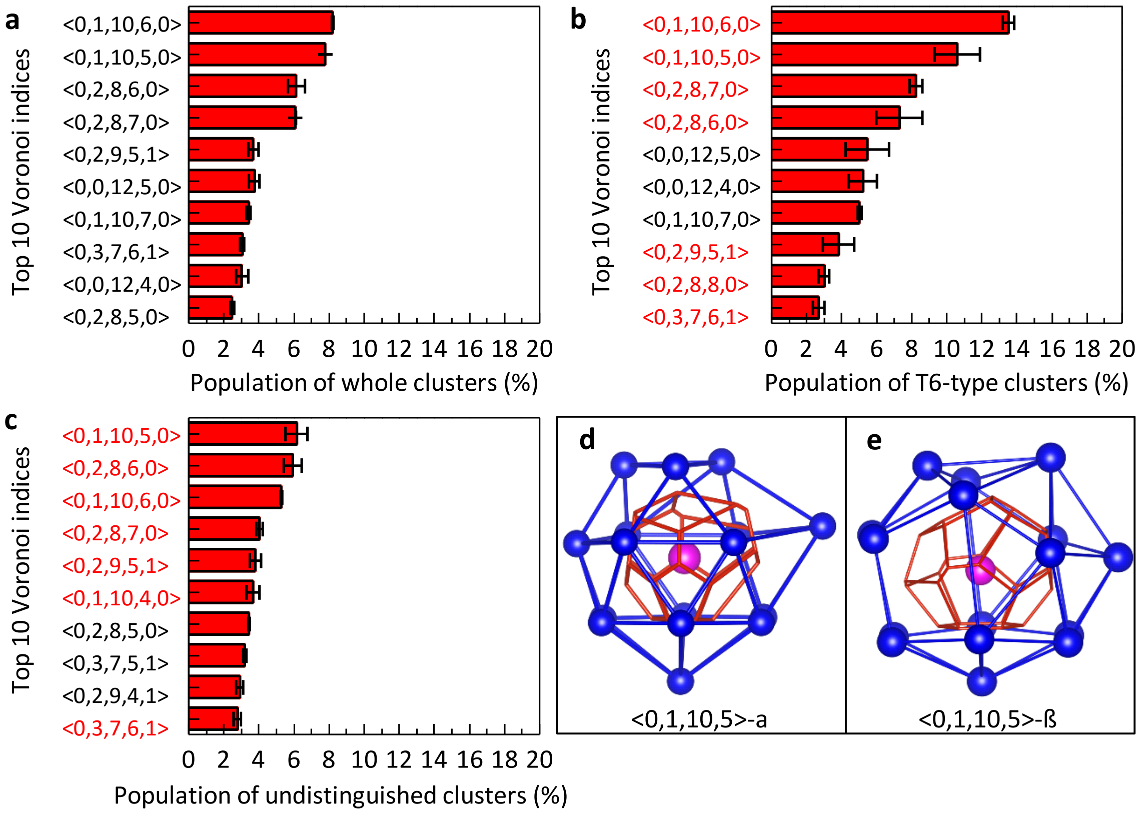

Supplementary Figure 4 | Voronoi tessellation analysis for Sm-centered clusters. (a) The top ten Voronoi polyhedra for the whole Sm-centered clusters; (b), the top ten Voronoi polyhedra for the Sm-centered clusters characterized as T6-type by our GA and cluster alignment methods described in the main text; (c) the top ten Voronoi polyhedra for the Sm-centered clusters that cannot be characterized by any known motifs, by the cluster alignment method. (d) and (e), Two polyhedra from GA-searched AlSm crystal structures both showing Voronoi index <0,1,10,5>. Common indices between the (b) and (c) top ten groups are highlighted in red.

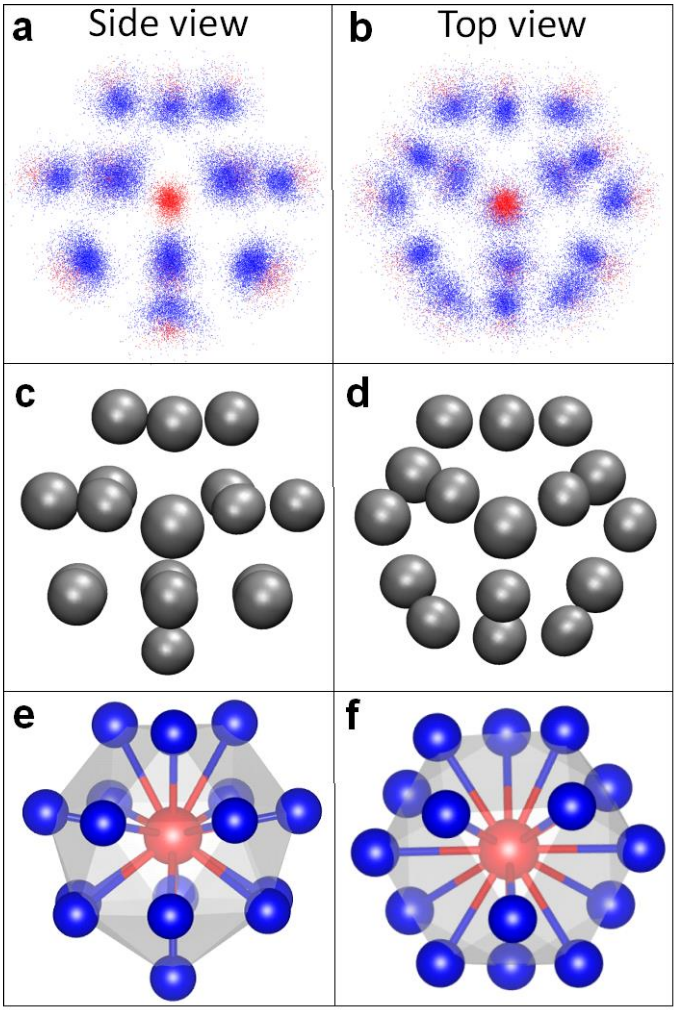

Supplementary Figure 5 | The validation of alignment analysis for T6-type clusters. To validate the structural order revealed by the alignment analysis, 3000 aligned clusters which are identified as T6 motif are superposed by the center atom. (a) and (b) show the side and top view for the configuration of superposed clusters. The red points represent Sm atoms, while blue points represent Al atoms. (c) and (d) are the side and top view for atomic-density contour plots corresponding to the atomic distribution in (a) and (b), respectively, obtained by the Gaussian smearing scheme explained in the Method section. (e) and (f) show the perfect T6 motif. One can see a good agreement between the common short range order of the identified clusters and the template motif.

Supplementary Note 4: X-ray diffraction measurements. The Al-10at.% Sm alloy was synthesized by arc melting high-purity elements (>99.9%) in a Ti-gettered Ar environment. After melting and flipping several times to assure homogeneity, the alloy was cast into a 6 mm mold. The cast ingot was then melted in an induction furnace and injection cast into a 1.6 mm mold to form rods that were placed in silica capillary tubes. The X-ray experiments were performed at Sector 6-ID-D of the Advanced Photon Source at Argonne National Laboratory. The samples were heated in furnace that was placed 347 mm upstream from a MAR CCD detector that was positioned off-axis to collect a higher *Q*-range. X-rays with an energy of 100 keV (λ = 0.1245 A) were utilized in the experiments, which were performed in transmission mode. The collected diffraction patterns were integrated using Fit2D software [9], and corrected for absorption, polarization, multiple scattering, and Compton scattering [10]. The total scattering functions, *S(Q)*, at the different temperatures were calculated according to

$$S(Q) = 1 + \frac{\left[I(Q) - \sum_{i=1}^{n} a_i \left| f_i(Q) \right|^2 \right]}{\left| \sum_{i=1}^{n} a_i f_i(Q) \right|^2}, \qquad (1)$$

where $Q = 4\pi \sin\theta/\lambda$, $I(Q)$ is the coherently scattered portion of the total intensity, a_i is the atomic proportion of each element, and $f_i(Q)$ is the *Q*-dependent scattering factor for each element.

Supplementary Note 5: Classification of the template cluster motifs. Cluster alignment algorithm was also used to check the similarity among the template motifs given in Fig. 2 of the main text. In Supplementary Fig. 6a, we connect any pair of motifs if the score of their mutual alignment is smaller than the cut-off value of 0.19. While the

motifs T4, T5, and T9 are isolated, the remaining cluster motifs show complex correlations among themselves.

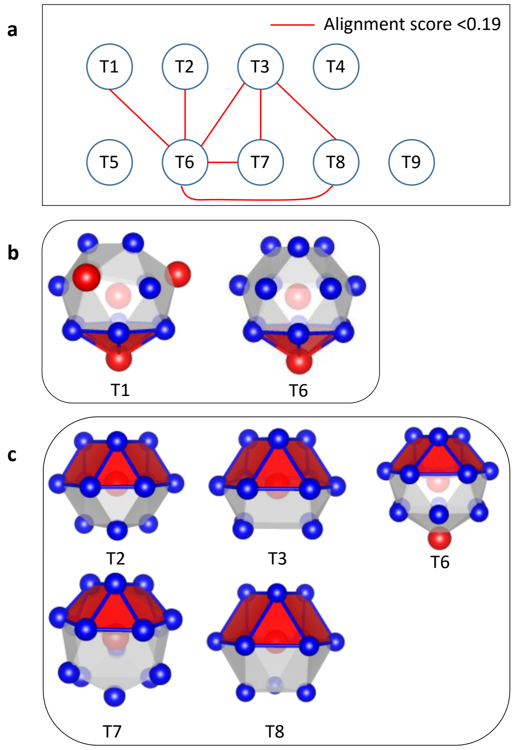

Supplementary Figure 6 | Classification of the template cluster motifs by the cluster alignment algorithm. (a) The cluster motifs are connected if their mutual alignment score is less than the cut-off value of 0.19. (b) T1 and T6 motifs share a common Al-Sm "hexagonal pyramid". (c) T2, T3, T6, T7 and T8 share a common "triangle and hexagon" packing of Al atoms.

After a closer inspection, one can identify some common features of this group: (1) T1 and T6 share a hexagonal pyramid at the bottom as shown in Supplementary Fig. 6b; (2) T2, T3, T6, T7 and T8 share a "triangle and hexagon" packing of Al atoms, which is characteristic of the A-B stacking in close packing structures as shown in Supplementary Fig. 6c. The genetic algorithm (GA) identified motif T6 is the most representative of this group since it's connected with all other motifs in this group in Supplementary Fig. 6a.

Therefore, only T6 is shown with three other well separated motifs (T4, T5 and T9) in Fig. 5b of the main text to classify as-extracted clusters.

Supplementary References

1. Lee, M., Lee, C. M., Lee, K. R., Ma, E. & Lee, J. C. Networked interpenetrating connections of icosahedra: Effects on shear transformations in metallic glass. *Acta. Mater.* **59**, 159-170 (2011).

2. Soklaski, R., Nussinov, Z., Markow, Z., Kelton, K. F. & Yang, L. Connectivity of icosahedral network and a dramatically growing static length scale in Cu-Zr binary metallic glasses. *Phys. Rev. B* **87**, 184203 (2013).

3. Zhang, Y. *et al.* Cooling rates dependence of medium-range order development in Cu64.5Zr35.5 metallic glass. *Phys. Rev. B* **91**, 064105 (2015).

4. Fang, X. W., Wang, C. Z., Yao, Y. X., Ding, Z. J. & Ho, K. M. Signature of Al11Sm3 fragments in undercooled Al90Sm10 liquid from ab initio molecular dynamics simulations. *J. Phys. Condens. Matter* **23**, 235104 (2011).

5. Fang, X. W., Wang, C. Z., Yao, Y. X., Ding, Z. J. & Ho, K. M. Competition between fcc and icosahedral short-range orders in pure and samarium-doped liquid aluminum from first principles. *Phys. Rev. B* **83**, 224203 (2011).

6. Gaskell, P. A new structural model for transition metal–metalloid glasses. *Nature* **276**, 484-485 (1978).

7. Miracle, D. B. A structural model for metallic glasses. *Nat. Mater.* **3**, 697-702 (2004).

8. Troadec, J., Gervois, A. & Oger, L. Statistics of Voronoi cells of slightly perturbed face-centered cubic and hexagonal close-packed lattices. *EPL* **42**, 167 (1998).

9. Hammersley, A., Svensson, S., Hanfland, M., Fitch, A. & Hausermann, D. Two-dimensional detector software: from real detector to idealised image or two-theta scan. *High Pressure Res.* **14**, 235-248 (1996).

10. Qiu, X., Thompson, J. W. & Billinge, S. J. PDFgetX2: a GUI-driven program to obtain the pair distribution function from X-ray powder diffraction data. *J. Appl. Crystallogr.* **37**, 678-678 (2004).
